# Impact of a district-wide health center strengthening intervention on healthcare utilization in rural Rwanda: Use of interrupted time series analysis

**DOI:** 10.1371/journal.pone.0182418

**Published:** 2017-08-01

**Authors:** Hari S. Iyer, Lisa R. Hirschhorn, Marie Paul Nisingizwe, Emmanuel Kamanzi, Peter C. Drobac, Felix C. Rwabukwisi, Michael R. Law, Andrew Muhire, Vincent Rusanganwa, Paulin Basinga

**Affiliations:** 1 Division of Global Health Equity, Brigham and Women’s Hospital, Boston, Massachusetts, United States of America; 2 Partners In Health/Inshuti Mu Buzima, Kigali, Rwanda; 3 Harvard T. H. Chan School of Public Health, Boston, Massachusetts, United States of America; 4 Department of Medical Social Sciences, Northwestern University Feinberg School of Medicine, Chicago, Illinois, United States of America; 5 Department of Global Health and Social Medicine, Harvard Medical School, Boston, Massachusetts, United States of America; 6 Partners In Health, Boston, Massachusetts, United States of America; 7 Centre for Health Services and Policy Research, The University of British Columbia, Vancouver, British Columbia, Canada; 8 Ministry of Health, Kigali, Rwanda; 9 Bill and Melinda Gates Foundation, Seattle, Washington, United States of America; Kenya Medical Research Institute - Wellcome Trust Research Programme, KENYA

## Abstract

**Background:**

Evaluations of health systems strengthening (HSS) interventions using observational data are rarely used for causal inference due to limited data availability. Routinely collected national data allow use of quasi-experimental designs such as interrupted time series (ITS). Rwanda has invested in a robust electronic health management information system (HMIS) that captures monthly healthcare utilization data. We used ITS to evaluate impact of an HSS intervention to improve primary health care facility readiness on health service utilization in two rural districts of Rwanda.

**Methods:**

We used controlled ITS analysis to compare changes in healthcare utilization at health centers (HC) that received the intervention (n = 13) to propensity score matched non-intervention health centers in Rwanda (n = 86) from January 2008 to December 2012. HC support included infrastructure renovation, salary support, medical equipment, referral network strengthening, and clinical training. Baseline quarterly mean outpatient visit rates and population density were used to model propensity scores. The intervention began in May 2010 and was implemented over a twelve-month period. We used monthly healthcare utilization data from the national Rwandan HMIS to study changes in the (1) number of facility deliveries per 10,000 women, (2) number of referrals for high risk pregnancy per 100,000 women, and (3) the number of outpatient visits performed per 1,000 catchment population.

**Results:**

PHIT HC experienced significantly higher monthly delivery rates post-HSS during the April-June season than comparison (3.19/10,000, 95% CI: [0.27, 6.10]). In 2010, this represented a 13% relative increase, and in 2011, this represented a 23% relative increase. The post-HSS change in monthly rate of high-risk pregnancies referred increased slightly in intervention compared to control HC (0.03/10,000, 95% CI: [-0.007, 0.06]). There was a small immediate post-HSS increase in outpatient visit rates in intervention compared to control HC (6.64/1,000, 95% CI: [-13.52, 26.81]).

**Conclusion:**

We failed to find strong evidence of post-HSS increases in outpatient visit rates or referral rates at health centers, which could be explained by small sample size and high baseline nation-wide health service coverage. However, our findings demonstrate that high quality routinely collected health facility data combined with ITS can be used for rigorous policy evaluation in resource-limited settings.

## Introduction

Health systems strengthening (HSS) interventions have become popular strategies to advance population health gains in low income countries [1–5]. Instead of focusing on disease-specific, or “vertical” programs, HSS interventions improve the platform of health service delivery across all health system components [1, 3]. While the evidence base of successful HSS interventions that improve processes and health outcomes is growing [5, 6], few evaluations have quantified the resulting changes in healthcare utilization, a critical step towards increasing coverage of health services [7, 8]. Understanding the link between investment in systems and uptake can inform health resource allocation and planning efforts in low income settings [9]. However, the cost of collecting novel data to measure the effect of HSS interventions presents a barrier to measuring impact and often results in inadequate evaluation.

An underutilized resource that could be used to address this issue is the wealth of routinely collected service utilization data produced by national health management information systems (HMIS) in low income countries [8, 10]. Analysis using HMIS leverages health systems time series data that are already being used for management and improvement, while allowing for evaluation designs informed by principles of causal inference [11]. A potential reason for limited use of HMIS for HSS evaluations is the perception that data quality is poor [12, 13], despite several positive results following data quality assessments of these systems in low income countries [14–16].

Rwanda, a small, hilly country in East Africa, has embraced the use of HMIS and is well positioned to take advantage of this resource. Rwanda has heavily invested in its Rwanda HMIS (RHMIS), which provides managers and policy makers with healthcare utilization data from all public health facilities in the country. Reports on utilization of outpatient and inpatient services are collected from registers by facility data staff at every public health facility in the country, aggregated at the district level, and are subsequently uploaded to a web-based central repository at the national level [17]. A recent national data quality assessment demonstrated high levels of completeness and internal consistency of RHMIS [15]. An accuracy assessment that sampled facility records across three rural districts found concordance between hard copy reports and electronic RHMIS reports to be 73.3% and concordance between facility registers and electronic RHMIS reports to be 70.6% [18]. Assessments of external validity comparing coverage estimates for family planning and 4 ANC visits in RHMIS to the Demographic and Health household Survey (DHS) conducted by RHMIS analysts were found to be comparable. Institutional delivery estimates were slightly lower in RHMIS compared to DHS in 2010 (57% vs. 69%) [19].

Rwanda has begun to leverage this resource in national evaluations of several health policies, including performance-based financing (PBF) [20], Community Health Worker (CHW) programs [21], and uptake of maternal and reproductive health services following implementation of Human Immunodeficiency Virus (HIV) control programs [22]. These data systems have allowed scientists and policymakers to describe improvements in health outcomes that have occurred in Rwanda over the past decade, which include steep reductions in under-five mortality [23–25], as well as increased coverage of antiretroviral therapy services [26] and maternal health services [27], suggesting improved access to a range of health services across the country [20, 28–31]. While providing important contributions to the health systems literature by documenting health gains in Rwanda, these studies largely relied on cross-sectional or pre-post designs without a control, and so have limited utility for causal inference. Other countries in the region including Uganda and Burundi have also used their national health information systems to assess the impact of national health care financing policies on service utilization [32, 33]. Basinga et al.’s evaluation of PBF relied on a randomized controlled trial design, and Falisse et al. employed a difference-in-difference analysis using two single time points for the intervention and control series in a PBF evaluation in Burundi, but few other analyses using routinely collected national data have leveraged the longitudinal data structure or the ability to sample control series from a national census.

In this manuscript, we evaluate the impact of a district-level HSS intervention implemented by the Rwanda Population Health Implementation and Training (PHIT) partnership in rural Rwanda on district-level health service utilization [34, 35]. Using RHMIS as our data source, we use five years of monthly health center-level time series data to conduct a propensity score matched controlled interrupted time series analysis to estimate the district-level impact of the PHIT HSS intervention on delivery rates, outpatient visits rates, and referral rates for high risk pregnancies. Controlled interrupted time series analysis allows for unbiased estimation of population level effects of an intervention, assuming that no other co-interventions occur at the same time as the primary intervention [36]. This evaluation will inform other global health researchers in Rwanda and elsewhere about the effectiveness of health systems interventions on increasing service uptake, and demonstrate the power of combining national HMIS time series data with counterfactual-based methods to allow causal conclusions to be drawn from HSS impact evaluations in global health.

## Methods

### Study setting, population, and intervention

Rwanda has a population of 10.5 million, 83% of whom live in rural areas [37]. In 2012, Rwanda had 748 public (government-run) health centers and 174 private health centers, with most care provided at public health centers (85–89%) [15]. The first facility point of contact for patients are public health centers, which provide primary care and maternal and child health services. Each of Rwanda’s 30 districts has roughly fifteen health centers, with the goal of every patient in Rwanda to be living within five kilometers of a health facility. Rwanda’s health system is also served by a network of CHW who administer treatments for childhood illnesses (community integrated management of childhood illness), or refer patients to health centers or hospitals for further care [21]. Health centers in Rwanda provide a minimum package of services that span promotional (child growth monitoring and community health insurance), preventive (vaccination, prenatal and postnatal care) and curative activities (child health care, uncomplicated deliveries, HIV, drug dispensation). These services are offered at all health centers in the country [31]. The CHW program was implemented between 2008 and 2011, and overlapped with the implementation of our HSS intervention.

In 2009, Partners In Health (PIH) and the Government of Rwanda (GOR) entered a partnership to implement a five-year district-wide HSS intervention in two rural districts of Rwanda serving a catchment population of 480,000, which lagged behind the rest of the country in terms of health and social indicators [27, 34]. These districts were chosen because PIH was already supporting the GOR through provision of technical and financial support to two district hospitals and seven health centers in these districts prior to the PHIT intervention. For this analysis, we use ITS to study the impact of the first component intervention that began in May 2010 and consisted of targeted instrumental support to PHIT health centers. This included a data-driven gap analysis to assess facility readiness among the fourteen health centers in the intervention area that had not received partnership support prior to the HSS intervention. Facility surveys were developed to ascertain dimensions of facility readiness, guided by the World Health Organization (WHO) health systems building block framework [9] and based on the Services Provision Assessment [38] and the annual health facility survey which was in use nationally at the start of the intervention. Core domains included infrastructure, human resources, monitoring and evaluation, and supplies with an emphasis on data utilization for decision-making. Partnership representatives met with health facility leadership to discuss prioritization of resource allocation based on survey results. Based on review of the results and these discussions, limited funds (average: 18,000 USD/health center) were transferred to intervention facilities to address prioritized gaps. Specific areas of focus varied by facility and ranged from investments in health center management, infrastructure renovations, medical equipment, salary support for additional health center staff, and social support for vulnerable patients resulting in overall improvement of facility readiness to provide care [35]. Following this work, additional district-wide interventions focused on further strengthening facility and CHW care quality and service utilization in the intervention area [39, 40].

### Study design

**Fig 1:**
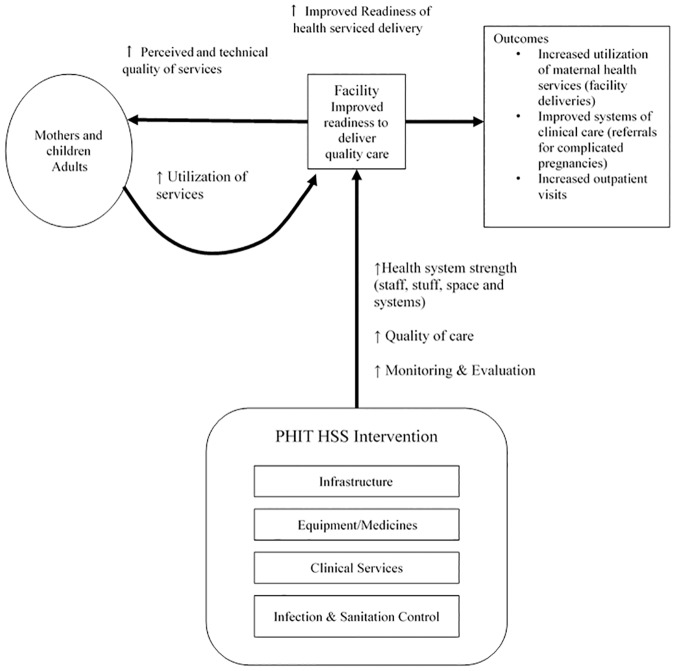
Rwanda Population Health Implementation and Training health systems strengthening evaluation conceptual framework.

We developed a conceptual framework describing the pathway from intervention to increased uptake of services among people living in the intervention catchment area (Fig 1). We hypothesized that the improved facility readiness to provide high quality care through strengthened infrastructure, supplies, staff and information systems would be recognized by community members who would be more likely to seek care at the intervention facilities and recommend use by others [35]. This framework was supported by research on factors associated with care seeking behavior have shown that availability of equipment and medicines leads to increased utilization [41], and that perceived poor quality of facilities can lead to reduced care seeking by pregnant women [42].

We framed our causal question as follows: what is the difference in level and monthly trend in mean health service utilization rates comparing observed rates from PHIT-supported health centers to the rates they would have had if they had not received the HSS intervention? Controlled interrupted time series operationalizes this question by modeling the counterfactual using segmented linear regression and the post-intervention level and trend from a control series [43].

There were a total of 21 health centers in the intervention districts, seven of which had received support from PIH prior to the PHIT HSS intervention. These seven health centers were excluded because we were interested in the impact of PHIT HSS on health centers which had never received any non-governmental support, leaving us with 14 PHIT intervention health centers [35]. Our control series contained 409 government-run health centers that were also providing services. We sought to limit our control series to public health centers because the staff, infrastructure and equipment at private health centers would not reflect those available at the government-run intervention health centers prior to HSS. We excluded 29 health centers due to incomplete reporting over the study period, and the remaining 380 (93%) were eligible for matching.

### Data source

RHMIS data were migrated from a SQL-server database to a DHIS2 platform in 2011. We merged data from the two databases to generate an analysis dataset spanning January 2008 through December 2012.

### Outcomes

Utilization of maternal health and outpatient visits were chosen *a priori* because most of the investments were directed at these services, through investments in improved maternity infrastructure, clinical mentoring and essential medicines and equipment for maternal and newborn care [35]. Using the RHMIS data, we constructed a dataset containing variables on maternal health (new antenatal care registrations, women with 4 standard antenatal care visits, facility deliveries, referrals for high risk pregnancy, family planning) outpatient visits, and child care (diphtheria-pertussis-tetanus (DPT) DPT1, DPT3, and BCG vaccination visits).

We converted these metrics to rates using population estimates from the Ministry of Health [37] and measured differences in the monthly number of facility deliveries per 1,000 women, the monthly number of referrals for high risk pregnancy per 10,000 women, monthly number of outpatient (OPD) visits per 1,000 catchment population, monthly number of DPT1 vaccination visits per 1,000 children 0–11 months, monthly number of DPT3 vaccination visits per 1,000 children 0–11 months, and monthly number of BCG vaccination visits per 1,000 children 0–11 months between the intervention health centers and propensity score matched health centers. Though our outcomes do not incorporate person-time, we refer to them as rates for succinctness. For analysis, monthly rates were aggregated by intervention group (PHIT versus propensity score matched non-intervention series).

### Statistical analysis

To account for differences in health center characteristics across intervention and control groups and select control facilities that were similar to intervention facilities at baseline with respect to health center characteristics, propensity scores were derived for each health center using multiple logistic regression models [44]. We limited the number of covariates included in the model due to the small number of intervention health centers (n = 14). We hypothesized that population density, outpatient visit rates, and delivery rates would be associated with success of the PHIT intervention and so we wanted to match intervention health centers to control health centers with similar characteristics at baseline. We chose to restrict the baseline period from January 2008 to December 2009 to provide two full years, and eligible control health centers to those that had no more than four missing HMIS reports during the baseline period. We failed to find evidence of association between delivery rates and treatment after adjusting for population density and outpatient rates, so our final model included population density (continuous, number of people per square kilometer in a catchment area sector), and four covariates that summarized the monthly average outpatient rate at each health center over six month intervals (January 2008-June-2008, July 2008-December 2008, January 2009-June 2009, July 2009-December 2009). To improve precision, each intervention health center was matched to up to ten control health centers, using a caliper match of ±0.05 propensity score units [45, 46]. We assessed the performance of the propensity score match by comparing the standardized difference in health service utilization between intervention and control health centers before and after applying the match [47, 48].

We used controlled interrupted time series analysis to study trends in healthcare utilization variables in the intervention group relative to the comparison group. We used the intervention date of May 2010 and fit time series models to test whether differences in changes in level or trend in indicators were statistically significant between the two groups. Controlled interrupted time series produces two main results of interest. The first is the difference in post-implementation change in level of mean outcome in the intervention relative to the control group, and the second is the difference in post-implementation trend in outcome in the intervention relative to the control group. These results are beta coefficient estimates produced by the time series models. For our study, we interpret these beta coefficients as 1) the difference in mean health service utilization rate from the pre-intervention to post-intervention period comparing the intervention facilities to control facilities, and 2) the difference in monthly change in service utilization rate from pre-intervention to post-intervention period comparing intervention facilities to control facilities. We plotted our results using fitted line segments to visualize these pre and post-intervention changes in level and trend by intervention group. The trend coefficient in our model allowed us to estimate any changes that would arise in our intervention over time, such as delayed improvements following the initial investments into the system.

We used generalized least squares (GLS) models including autocorrelation terms for both moving average or autoregressive processes that were assessed independently for each outcome as described by Wagner et al. [43]. Lag terms were determined using Durbin-Watson tests and autocorrelation and partial autocorrelation plots. We used a GLS model with an autocorrelation lag term of 4 and a moving average lag term of 4 to analyze trends in delivery rates. For OPD, we used a GLS model with a moving average lag term of 1. Finally, we used a GLS model with an autocorrelation lag term of 1 and a moving average lag term of 1 for analysis of rates of referral for high risk pregnancy. We chose to control for seasonality by including a seasonal dummy variable corresponding to three month quarters (January to March, April to June, July—September, and October to December) for facility deliveries and OPD [49]. This seasonality dummy was not included in the model for high risk pregnancy because we failed to find any statistically significant associations between seasonality and trends in this variable. We also conducted *post hoc* tests for effect modification of the post-implementation effect on healthcare utilization on the multiplicative scale by season in the April-June quarter using a multiplicative interaction term. Analysis was conducted SAS v. 9.4 (propensity score matching) and R v. 3.1.0 (controlled interrupted time series) and. Full details regarding the statistical models are provided in the supplementary appendix (S1 File). Statistical significance was assessed at the 0.05 level, and results are presented as point estimates and 95% confidence intervals (95% CI).

## Results

### Baseline characteristics

We present baseline characteristics of the intervention and control facilities in Table 1. Intervention facilities covered a much smaller total population than control facilities (254,656 v. 8,474,422) and were distributed over much smaller geographic area. Intervention facilities had slightly fewer monthly new ANC registrations on average during the baseline period of 2008–2009 compared to comparison facilities (median: 44 v. 62), and also fewer monthly outpatient visits (median: 945 v. 1326) compared to comparison facilities. Monthly facility deliveries over the baseline period were similar for both groups. Both intervention and comparison facilities had few referrals for high-risk pregnancies.

**Table 1:** Baseline health center characteristics of intervention and control facilities.

	Pre-match						Post-match					
	PHIT Intervention		Eligible Control Health Centers		Absolute difference	Standardized difference	PHIT Intervention		Propensity Score Matched Controls		Absolute difference	Standardized difference
Total catchment population	254,656		8,474,422				238,693		1,675,011			
Districts	2		29				2		25			
Number of facilities	14		380				13		86			
Population density (people/km^2^) (median, IQR)	379	[279–408]	495	[392–600]	-116	-1.06	315	[279–379]	390	[307–478]	-75	-0.42
Baseline utilization (2008–2009), median, [IQR*]												
Monthly new ANC registrations	44	[25–65]	62	[41–91]	-18	-0.59	46	[25–67]	54	[37–75]	-8	0.43
Monthly facility-based deliveries	26	[17–41]	32	[20–45]	-6	-0.26	28.75	[17–43]	28.5	[18–40]	0.25	-0.11
Monthly referrals for high risk pregnancies	0	[0–1]	1	[0–3]	-1	-0.56	0	[0–1]	1	[0–3]	-1	-0.08
Monthly outpatient visits	945	[596–1,325]	1,326	[875–1,964]	-381	-0.64	894	[569.5–1301.5]	1090	[741–1458]	-196	-1.81

*IQR = interquartile range

Propensity score matching resulted in 13 intervention health centers matched to 86 control health centers on population density and monthly outpatient visits over four six-month intervals between January 2008 –December 2009. Parameter estimates and kernel density plots showing results of the matching are presented in the supplementary content (S1 Fig, S1 Table). Propensity score matching yielded improved balance with respect to facility-based deliveries (standardized difference post-matching compared to pre-matching: -0.11 v. -0.26), population density (standardized difference post-matching compared to pre-matching: -0.42 v. -1.06), and referrals for high risk pregnancy (standardized difference comparing post- to pre-matching: -0.08 v. -0.56). The standardized difference for outpatient visits increased (-1.81 v. -0.64), though the absolute difference between PHIT and control facilities decreased (-196 v. -381) after matching.

### Facility-based deliveries

**Fig 2:**
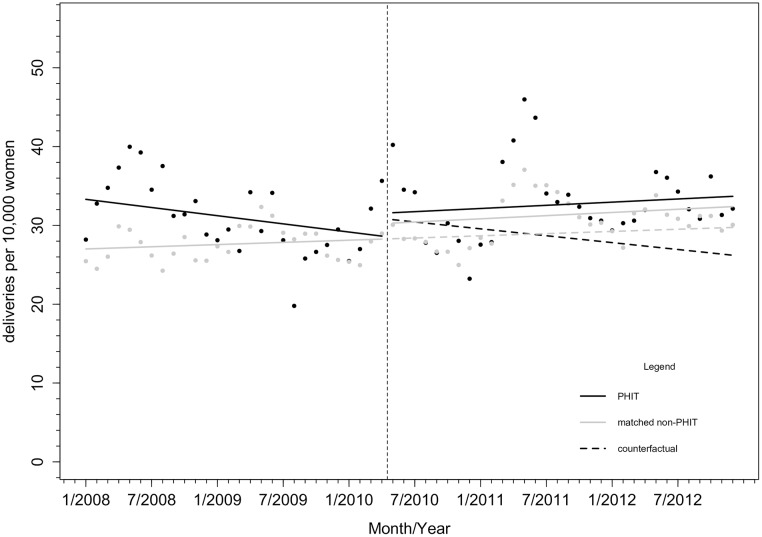
Monthly facility delivery rates (count per 10,000 women in the population) in PHIT health facilities (n = 13) or comparison propensity score matched facilities (n = 86). Notes: Vertical line indicates start of PHIT HSS intervention in May 2010.

As shown in Fig 2, the average monthly facility-based delivery rate was higher (6.61/10,000, 95% CI: [1.54, 11.67]), and the trend in monthly facility-based delivery rate was declining (-0.21/10,000, 95% CI: [-0.51, 0.08]) in PHIT facilities relative to comparison facilities over the period prior to HSS. After implementation of HSS, there was a small immediate increase in the level (0.67/10,000, 95% CI: [-5.40, 6.74]) and trend in monthly facility delivery rates (0.21/10,000, 95% CI: [-0.19, 0.62]). Following HSS, PHIT facilities experienced significantly higher monthly delivery rates during the April-June season than comparison facilities (3.19/10,000, 95% CI: [0.27, 6.10]). In 2010, this represented an additional 4.1 deliveries/10,000 women in PHIT health centers (13% relative increase) compared to the rates in comparison health centers, and in 2011, this represented an additional 6.8 deliveries/10,000 women in PHIT compared to non-PHIT facilities (23% relative increase). Parameter estimates are reported in S2 Table.

### Referrals for high-risk pregnancies

**Fig 3:**
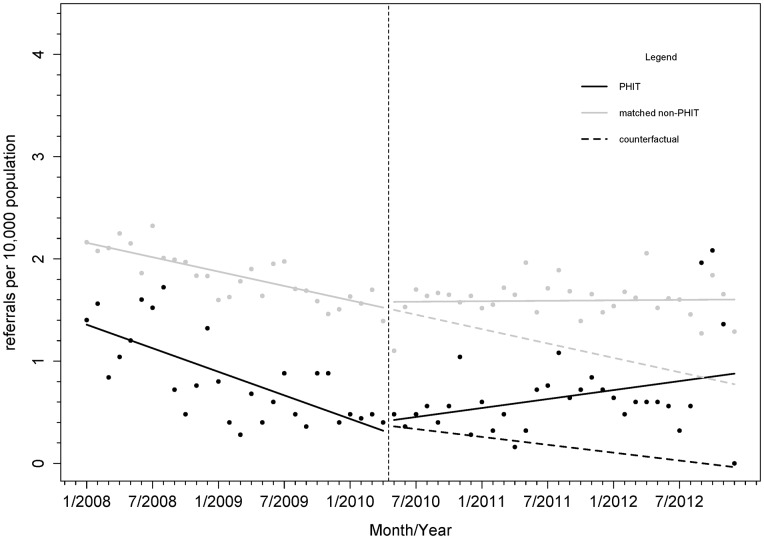
Monthly rates of referral for high risk pregnancy (count per 10,000 women in the population) in PHIT health facilities (n = 13) or comparison propensity score matched facilities (n = 86). Notes: Vertical line indicates start of PHIT HSS intervention in May 2010.

Over the period prior to HSS, the mean rate of high-risk pregnancy referrals at intervention facilities was lower than at control facilities (-0.82/10,000, 95% CI: [-1.26, -0.39]) (Fig 3). Mean referral rates at intervention facilities were 0.035/10,000 higher than the rates in comparison facilities in the period after HSS implementation began (95% CI: [-0.51, 0.58]). The trend in monthly rate of high-risk pregnancies referred increased slightly in intervention facilities compared to the control following the start of the intervention (0.027/10,000, 95% CI: [-0.007, 0.06]). We also found increasing trend in monthly referral rates (0.024/10,000, 95% CI: [-0.0002, 0.049]) in the control facilities following implementation of our HSS intervention. Parameter estimates are reported in S3 Table.

### Outpatient visits

**Fig 4:**
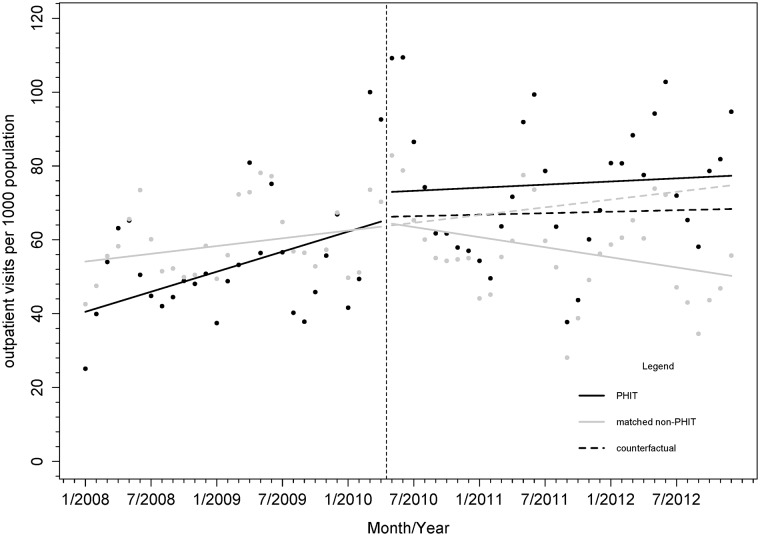
Monthly rates of outpatient visits (count per 1,000 catchment population) in PHIT health facilities (n = 13) or comparison propensity score matched facilities (n = 86). Notes: Vertical line indicates start of PHIT HSS intervention in May 2010.

At baseline, the average outpatient visit rate was 26% lower in PHIT health centers compared to comparison health centers (13.3/1,000 people, 95% CI: [-29.1, 2.6]). There were no major differences in level or trend in mean monthly outpatient visit rates in PHIT health centers relative to comparison facilities following implementation of HSS (Fig 4). While there was an immediate 27% increase (18.0 visits per 1,000 people per month) in the rate of outpatient visits in intervention facilities compared to control facilities in the period following HSS, (6.64/1,000, 95% CI: [-13.52, 26.81]), this did not reach statistical significance. Over time there was a decline in the trend in monthly mean outpatient visit rate in the comparison facilities (-0.81/1,000, 95% CI: [-1.69, 0.08]). There was a slight increasing trend in monthly mean outpatient rates following implementation of HSS in the intervention facilities compared to the comparison facilities, but this was not statistically significant (0.073/1,000, 95% CI: [-1.18, 1.32]). Parameter estimates are reported in S4 Table.

### Other indicators

We found no significant differences in level or trend for rates of childhood vaccinations (DTP1, DTP3, BCG), new antenatal care registrations, or women with 4 standard antenatal care visits in the intervention facilities relative to comparison facilities (S5, S6, S7, S8 and S9 Tables).

## Discussion

We present results from one of the first evaluations of an HSS intervention on health service utilization conducted using controlled interrupted time series and routinely collected HMIS data. Our findings showed that the impact of the PHIT HSS intervention on service utilization was constrained to facility-based delivery rates. We failed to find evidence that the HSS intervention resulted in increases in monthly outpatient visit rates or rates of referral for high risk pregnancy over and above those found in comparison facilities. For deliveries, we found that the increase in rates following the PHIT HSS intervention was restricted from the April to June season. For outpatient visit rates, even though we did not find evidence of a significant increase in level or trend in PHIT compared to control health centers following HSS, the level and trend in PHIT health centers remained higher than in control over the post-implementation period despite having lower rates at baseline.

Facility-based deliveries were declining in the intervention health centers prior to HSS, with significant increase in mean delivery rates during summer months over the implementation period. Prior to the intervention, PIH had provided support to the two district hospitals and only seven health centers (excluded from the intervention group) [35]. One possible explanation of the decline in the pre-HSS period was patients choosing to go to health centers that were already receiving non-governmental supports within the intervention districts over their local health centers. There is growing evidence that women will indeed bypass facilities which they perceive as providing worse quality of care [42]. It is possible that positive messages about improved quality following the intervention combined with better access to health centers for deliveries during April-June may have driven the increase in delivery rates. The April-June season overlaps with the end of the short rainy season and beginning of the long dry season in Rwanda, during which roads improve and therefore accessibility to health centers increases.

Following the health center strengthening intervention, implementation of a quality of care intervention began that could also have led to increased utilization of maternal health services began in May 2011 [40]. Improving service readiness at multiple facilities in the intervention area would have allowed more women to access health services closer to their homes—a predictor that has found to be associated with facility-based deliveries in other studies in Rwanda [50]. While previous studies have shown that health facility-level improvements focused just on supplies and other readiness factors do not always lead to increases in utilization in other developing countries [51–53], we demonstrate that in Rwanda, these improvements could have led to increased utilization for maternal health services. We attribute these increases to a comprehensive approach to HSS—our intervention was guided by all six of the WHO Building Blocks and included a strong focus on the quality of care delivered to address multiple components critical to responsive health service delivery [34, 35].

We did not find significant increases in referral rates in intervention health centers compared to control health centers following HSS. Since our health center strengthening intervention also included the provision of ambulances and materials to strengthen communication between health centers and hospitals, we hypothesized that these improvements could have led to increases in referrals for high-risk pregnancies. However, our integrated approach to improving health center readiness through infrastructure renovations, availability of equipment and supplies, staffing and social support could have also decreased barriers to care at intervention facilities. Increased receipt of maternal care at intervention health centers could have led to reductions in management of complicated deliveries through better care and skills. Our finding of suggestive trends in control areas post-HSS suggests that some other national co-intervention may have occurred concurrently with the PHIT intervention, thus mitigating our ability to detect additional improvement.

We failed to find significant increases in outpatient visit rates in intervention relative to the comparison health centers. Our intervention occurred during a period of rapid change in Rwanda’s health system [24, 25]. Many national policies to expand access to facility-based care and decentralize decision-making power were introduced in the years preceding the intervention and during the intervention [54]. We saw suggestion of decreasing trends in monthly outpatient visit rates in control health centers which suggests that this roll-out of community-based treatment of children under-five may have decreased use of services at facilities in the intervention and comparison areas. This hypothesis is supported by a recent analysis of community-based integrated community case management of childhood illness in Rwanda found decreases in facility utilization of under-5 services over the period of implementation (between January 2010 and December 2011) [21]. CHW strengthening was an explicit component of our broader health system intervention and may have been implemented with greater intensity in our districts compared to others, yielding differing patterns of healthcare utilization with regards to outpatient visits [34]. Given that implementation of this national policy occurred alongside our intervention, it is possible that it may have attenuated increases in facility-based outpatient visits during our study period.

The impact of various national policies to increase demand for maternal and child health services in Rwanda has been described in the literature [20, 28–31]. Researchers have argued that the collective impact of decentralization of health policy decision-making, community-based health insurance and the introduction of PBF have led to higher quality of services and increased utilization of public health services across the country, though only the PBF evaluation used a randomized controlled trial design. Further analyses have suggested that the benefits of these interventions have allowed the poorest patients in Rwanda to increasingly access health services over time [20, 30], meaning that the additional improvement attributable to the PHIT HSS intervention would be challenging to measure. We did not see any differences in healthcare utilization at intervention compared to non-intervention health centers across a number of services, many of which started at very high coverage rates prior to implementation of our HSS intervention. Antenatal care coverage and vaccination coverage were roughly 90% nation-wide prior to implementation of our HSS intervention [27], leaving little room for improvement over the study period.

Few evaluations exist to estimate the impact of HSS interventions to improve district facility readiness on service utilization, and fewer still are conducted using designs that allow causal effects to be estimated. Our study provides findings from an evaluation of such an intervention and shows that even in the context of national policies aimed at increasing utilization, facility deliveries in intervention health centers increased relative to control health centers in specific seasons. We would expect that in countries where access to health services is more limited and fewer national health financing schemes are in place to encourage demand, a similar intervention could yield greater increases in healthcare utilization. Health systems researchers in northern Uganda found increases in facility deliveries following a combined community-level and health facility-level intervention that occurred between January 2010 and September 2011 to improve quality of services, though they presented results from their intervention area only [55].

Our study had several limitations. First, while our data source has been shown to have strong internal validity [15], full assessments of reliability and accuracy of RHMIS have yet to be conducted. However, preliminary assessments of external validity comparing RHMIS to DHS estimates for coverage indicators show little discordance [19]. Furthermore, in order to bias our results, RHMIS data quality would have had to have changed differentially at the same time as the HSS intervention between intervention and control areas. The electronic system transitioned from a French SQL-based database to an English DHIS2 database in 2012, which limited variables to those that were consistent across systems, though this affected both intervention and control facilities. We were limited in our ability to match our intervention health centers to control health centers using propensity scores due to small sample size for our intervention group and lack of detailed information on health center characteristics in all public health centers in Rwanda. Further limitations of this analysis include a lack of data on contextual factors associated with health centers that could help explain these findings. Use of geospatial analysis and contextual information at the health center and district level would provide additional information on possible mechanisms through which the intervention was successful or not. Future studies should account for district-level and health center-level interventions in the design, and power their studies to estimate effects at each level. These results are restricted to public health centers; since most care in Rwanda is provided at public facilities, we expect that bias due to differential increases and use of private health centers post-HSS is limited. Our quasi-experimental, longitudinal design controls for the impact of any national co-interventions that were rolled out across the country during the study period, strengthening the validity of our findings. Our HSS intervention was heterogeneous, with allocation of funds towards strengthening health systems building blocks tailored to specific needs of different health centers, making it difficult to attribute impact to a specific intervention component. Since we used aggregate data for our analysis, we cannot assess the impact of the intervention on individual patients to determine whether we successfully targeted the most vulnerable in our catchment populations.

Strengths of this analysis include use of five years of monthly time series HMIS data to allow modeling of counterfactuals following our HSS intervention, and use of propensity score matching to simulate exchangeability of intervention and comparison facilities by matching on baseline trend and health center covariates. Since the HSS intervention start date is clear, and control time series were included to account for other co-interventions that might influence utilization, we are unlikely to have introduced systematic bias in our analysis.

In summary, we evaluated the impact of a heterogeneous HSS intervention on health service utilization in rural Rwanda using routinely collected HMIS data. We found that facility delivery rates increased post-HSS in intervention compared to non-intervention health centers, though this increase was restricted to the April-June quarter in years following HSS, though changes in other patterns of utilization were limited. This example demonstrates how routinely collected HMIS data in Sub-Saharan Africa can be used for quasi-experimental evaluation designs. Time series data allow researchers to move away from pre-post and cross-sectional evaluation designs to quasi-experimental designs using counterfactuals. In addition to informing program implementation and providing useful estimates of impact for health providers, such analyses also encourage strengthened information systems and national engagement in policy research [8]. This manuscript involved collaboration between academic, non-governmental and governmental scientists, and was supported through training funds to develop capacity in Rwanda to study national policies using interrupted time series analysis and HMIS data. We hope other implementers and policy researchers in low and middle income countries will be encouraged by such studies to use their national data sources for implementation and policy research. Further studies using these methods are needed to estimate effects of HSS interventions have on utilization and population health outcomes.

## Supporting information

S1 File Supplementary statistical appendix. (DOCX)

S1 Table Results of logistic regression model to estimate propensity scores among intervention (n = 14) and control (n = 380) health centers in Rwanda. (DOCX)

S2 Table Parameter estimates for monthly delivery rate generalized least squares model following a health center strengthening intervention in 99 propensity score matched health centers from January 2008 to December 2012. (DOCX)

S3 Table Parameter estimates for monthly high risk pregnancy referral rate generalized least squares model following a health center strengthening intervention in 99 propensity score matched health centers from January 2008 to December 2012. (DOCX)

S4 Table Parameter estimates for monthly outpatient visit rate generalized least squares model following a health center strengthening intervention in 99 propensity score matched health centers from January 2008 to December 2012. (DOCX)

S5 Table Parameter estimates for monthly 1^st^ ANC registration rate generalized least squares model following a health center strengthening intervention in 99 propensity score matched health centers from January 2008 to December 2012. (DOCX)

S6 Table Parameter estimates for monthly 4 standard ANC visits rate generalized least squares model following a health center strengthening intervention in 99 propensity score matched health centers from January 2008 to December 2012. (DOCX)

S7 Table Parameter estimates for monthly BCG vaccination rate generalized least squares model following a health center strengthening intervention in 99 propensity score matched health centers from January 2008 to December 2012. (DOCX)

S8 Table Parameter estimates for monthly DTP1 vaccination rate generalized least squares model following a health center strengthening intervention in 99 propensity score matched health centers from January 2008 to December 2012. (DOCX)

S9 Table Parameter estimates for DTP3 vaccination rate generalized least squares model following a health center strengthening intervention in 99 propensity score matched health centers from January 2008 to December 2012. (DOCX)

S1 Fig Kernel density plot for propensity scores in PHIT matching analysis. (TIF)

## References

[1] Frenk J. (2010) The global health system: Strengthening national health systems as the next step for global progress. *PLoS Med* 7(1): e1000089 doi: 10.1371/journal.pmed.1000089 20069038 10.1371/journal.pmed.1000089 PMC2797599

[2] Bennett S, Agyepong IA, Sheikh K, Hanson K, Ssengooba F, Gilson L. (2011) Building the field of health policy and systems research: An agenda for action. *PLoS Med* 8(8): e1001081 doi: 10.1371/journal.pmed.1001081 21918641 10.1371/journal.pmed.1001081 PMC3168867

[3] Bassett MT, Gallin EK, Adedokun L, Toner C. (2013) From the ground up: strengthening health systems at district level. *BMC Health Services Research* 13(Suppl 2):S2. 10.1186/1472-6963-13-S2-S2 PMC3668220 23819479

[4] Sherr K, Requejo JH, Basinga P. (2013) Implementation research to catalyze advances in health systems strengthening in sub-Saharan Africa: the African Health Initiative. *BMC Health Services Research* 13(Suppl 2):S1. 10.1186/1472-6963-13-S2-S1 PMC3668282 23819761

[5] Hafner T, Shiffman J. (2013) The emergence of global attention to health systems strengthening. *Health Policy Plan* 28: 41–50 doi: 10.1093/heapol/czs023 22407017 10.1093/heapol/czs023

[6] Bennett S, Adam T, Zarowsky C, Tangcharoensathien V, Ranson K, Evans T, et al (2008) From Mexico to Mali: Progress in health policy and health systems research. *Lancet* 372: 1571–78 doi: 10.1016/S0140-6736(08)61658-X 18984191 10.1016/S0140-6736(08)61658-X

[7] Adam T, Hsu J, de Savigny D, Lavis JN, Rottingen J, Bennett S. (2012) Evaluating health systems strengthening interventions in low-income and middle-income countries: are we asking the right questions? *Health Policy and Planning* 27(Suppl. 4):iv9–iv19. 23014156 10.1093/heapol/czs086

[8] Wagenaar BH, Sherr K, Fernandes Q, Wagenaar AC. (2015) Using routine health information systems for well-designed health evaluations in low- and middle income countries. *Health Policy Plan* 2015 4 16. pii: czv029. 10.1093/heapol/czv029 PMC4751224 25887561

[9] World Health Organisation and Alliance for Health Policy and Systems Research. (2009) Systems Thinking: for Health Systems Strengthening In Systems Thinking: For Health Systems Strengthening. Edited by de Savigny D, Taghreed A. Geneva: World Health Organisation and Alliance for Health Policy and Systems Research; 2009

[10] Binagwaho A, Scott KW. (2015) Improving the world’s health through the post-2015 development agenda: Perspectives from Rwanda. *International Journal of Health Policy and Management* 4(4), p.203 doi: 10.15171/ijhpm.2015.46 25844381 10.15171/ijhpm.2015.46 PMC4380561

[11] Murray CJ, Lopez AD. (2010) Production and analysis of health indicators: the role of academia. *PLoS Med* 7(11), p.e1001004 doi: 10.1371/journal.pmed.1001004 21151347 10.1371/journal.pmed.1001004 PMC2994663

[12] Mate KS, Bennett B, Mphatswe W, Barker P, Rollins N. (2009) Challenges for routine health system data management in a large public programme to prevent mother-to-child HIV transmission in South Africa. *PLoS One* 4: e5483 doi: 10.1371/journal.pone.0005483 19434234 10.1371/journal.pone.0005483 PMC2677154

[13] Maokola W, Willey BA, Shirima K, Chemba M, Armstrong Schellenberg JRM, Mshinda H, et al (2011) Enhancing the routine health information system in rural southern Tanzania: successes, challenges and lessons learned. *Trop Med Int Health* 16: 721 30. doi: 10.1111/j.1365-3156.2011.02751.x 21395928 10.1111/j.1365-3156.2011.02751.x

[14] Gimbel S, Micek M, Lambdin B, Lara J, Karagianis M, Cuembelo F, et al (2011) An assessment of routine primary care health information system data quality in Sofala Province, Mozambique. *Popul Health Metr* 9:12 doi: 10.1186/1478-7954-9-12 21569533 10.1186/1478-7954-9-12 PMC3112390

[15] Nisingizwe MP, Iyer HS, Gashayija M, Hirschhorn LR, Amoroso C, Wilson R, et al (2014) Toward utilization of data for program management and evaluation: quality assessment of five years of health management information system data in Rwanda. *Glob Health Action* 7: 25829 http://dx.doi.org/10.3402/gha.v7.25829 10.3402/gha.v7.25829 PMC4238898 25413722

[16] Wagenaar BH, Gimbel S, Hoek R, Pfeiffer J, Michel C, Manuel JL, et al (2015) Effects of a health information system data quality intervention on concordance in Mozambique: Time-series analyses from 2009–2012. *Population Health Metrics* 13(1), p.1. 25821411 10.1186/s12963-015-0043-3 PMC4377037

[17] RTI International. (2006). Rwanda HMIS Assessment Report. Research Triangle Park: RTI International pp. 23–24.

[18] Innocent K, Onzima RAD, Katongole SP and Govule P. (2016) Quality and use of routine healthcare data in selected districts of eastern province of Rwanda. *Int J Public Health* 4(2): 5–13.

[19] Rwanda Ministry of Health. (2012). Rwanda Health Statistics Booklet 2011. Kigali: Rwanda Ministry of Health p. 26.

[20] Basinga P, Gertler PJ, Binagwaho A, Soucat ALB, Sturdy J, Vermeersch CMJ. (2011) Effect on maternal and child health services in Rwanda of payment to primary health-care providers for performance: An impact evaluation. *Lancet* 377(9775): 1421–1428. doi: 10.1016/S0140-6736(11)60177-3 21515164 10.1016/S0140-6736(11)60177-3

[21] Mugeni C, Levine AC, Munyaneza RM, Mulindahabi E, Cockrell HC, Glavis-Bloom J, et al (2014) Nationwide implementation of integrated community case management of childhood illness in Rwanda. *Global Health*: *Science and Practice* 2(3): 328–341. 10.9745/GHSP-D-14-00080 PMC4168626 25276592

[22] Price JE, Leslie JA, Welsh M, Binagwaho A (2009) Integrating HIV clinical services into primary health care in Rwanda: a measure of quantitative effects. *AIDS care* 21(5), pp.608–614. doi: 10.1080/09540120802310957 19444669 10.1080/09540120802310957

[23] Logie DE, Rowson M, Ndagije F. (2008) Innovations in Rwanda's health system: Looking to the future. *Lancet* 372 9634: 256–261. doi: 10.1016/S0140-6736(08)60962-9 18619670 10.1016/S0140-6736(08)60962-9

[24] Binagwaho A, Farmer PE, Nsanzimana S, Karema C, Gasana M, Ngirabega JD, et al (2014) Rwanda 20 years on: Investing in life. *Lancet* 384: 371–75 doi: 10.1016/S0140-6736(14)60574-2 24703831 10.1016/S0140-6736(14)60574-2 PMC4151975

[25] Farmer PE, Nutt CT, Wagner CM, Sekabaraga C, Nuthulaganti T, Weigel JL, et al (2013) Reduced premature mortality in Rwanda: lessons from success. *BMJ* 346: f65 doi: 10.1136/bmj.f65 23335479 10.1136/bmj.f65 PMC3548616

[26] Nsanzimana S, Ruton H, Lowrance DW, Cishahayo S, Nyemazi JP, Muhayimpundo R, et al (2012) Cell phone-based and internet-based monitoring and evaluation of the national antiretroviral treatment program during rapid scale-up in Rwanda: TRACnet, 2004–2010. *JAIDS* 59(2): e17–e23 doi: 10.1097/QAI.0b013e31823e2278 22067668 10.1097/QAI.0b013e31823e2278

[27] National Institute of Statistics of Rwanda and Macro International Inc. Rwanda Demographic and Health Survey (2010). Calverton: Macro International Inc, 2012

[28] Bucagu M, Kagubare JM, Basinga P, Ngabo F, Timmons BK, Lee AC. (2012) Impact of health systems strengthening on coverage of maternal health services in Rwanda, 2000–2010: A systematic review. *Reproductive Health Matters* 20(39): 50–61. doi: 10.1016/S0968-8080(12)39611-0 22789082 10.1016/S0968-8080(12)39611-0

[29] Saksena P, Antunes AF, Xu K, Musango L, Carrin G. (2011) Mutual health insurance in Rwanda: Evidence on access to care and financial risk protection. *Health Policy* 99(3): 203–209. doi: 10.1016/j.healthpol.2010.09.009 20965602 10.1016/j.healthpol.2010.09.009

[30] Sekabaraga C, Diop F, Soucat A. (2011) Can innovative health financing policies increase access to MDG-related services? Evidence from Rwanda. *Health Policy and Planning* 26 (Suppl 2): ii52–ii62. 22027920 10.1093/heapol/czr070

[31] Lu C, Chin B, Lewandowski JL, Basinga P, Hirschhorn LR, Hill K, et al (2012) Towards universal health coverage: An evaluation of Rwanda *Mutuelles* in its first eight years. *PLoS One* 7(6): e39282 doi: 10.1371/journal.pone.0039282 22723985 10.1371/journal.pone.0039282 PMC3377670

[32] Nabyonga J, Desmet M, Karamagi H, Kadama PY, Omaswa FG, Walker O. (2005) Abolition of cost-sharing is pro-poor: evidence from Uganda. *Health Policy and Planning* 20(2), pp.100–108. doi: 10.1093/heapol/czi012 15746218 10.1093/heapol/czi012

[33] Falisse JB, Ndayishimiye J, Kamenyero V, Bossuyt M. (2014) Performance-based financing in the context of selective free health-care: an evaluation of its effects on the use of primary health-care services in Burundi using routine data. *Health Policy and Planning*, p.czu132. 10.1093/heapol/czu132 25533992

[34] Drobac PC, Basinga P, Condo J, Farmer PE, Finnegan KE, Hamon JK, et al (2013) Comprehensive and integrated district health systems strengthening: The Rwanda Population Health Implementation and Training (PHIT) Partnership. *BMC Health Services Research* 13(Suppl 2):S5 10.1186/1472-6963-13-S2-S5 PMC3668243 23819573

[35] Iyer HS, Kamanzi E, Mugunga JC, Finnegan K, Uwingabiye A, Shyaka E, et al (2015) Improving district facility readiness: A 12 month evaluation of a data-driven health systems strengthening intervention in rural Rwanda. *Glob Health Action* 8: 28365 http://dx.doi.org/10.3402/gha.v8.28365 10.3402/gha.v8.28365 PMC4490804 26140729

[36] Bernal JL, Cummins S, Gasparrini A. (2016) Interrupted time series regression for the evaluation of public health interventions: A tutorial. *International Journal of Epidemiology* 2016: 1–8. doi: 10.1093/ije/dyw098 27283160 10.1093/ije/dyw098 PMC5407170

[37] Republic of Rwanda National Institute of Statistics. (2012). 2012 Population and Housing Census. Kigali: Rwanda Ministry of Health. Annex 2.

[38] The Demographic and Health Surveys Program. Service Provision Assessments. [online] http://dhsprogram.com/What-We-Do/Survey-Types/SPA.cfm [Accessed: 17 Mar. 2016].

[39] Magge H, Anatole M, Cyamatare FR, Mezzacappa C, Nkikabahizi F, Niyonzima S, et al (2014) Mentoring and quality improvement strengthen integrated management of childhood illness implementation in rural Rwanda. *Archives of Disease in Childhood*: Published Online First: [12-May-2014] doi: 10.1136/archdischild-2013-305863 24819369 10.1136/archdischild-2013-305863

[40] Anatole M, Magge H, Redditt V, Karamaga A, Niyonzima S, Drobac P, et al (2013) Nurse mentorship to improve the quality of health care delivery in rural Rwanda. *Nurs Outlook* 61(3):137–44. doi: 10.1016/j.outlook.2012.10.003 23164530 10.1016/j.outlook.2012.10.003

[41] Kruk ME, Paczkowski M, Mbaruku G, de Pinho H, Galea S. (2009) Women's preferences for place of delivery in rural Tanzania: A population-based discrete choice experiment. *American Journal of Public Health* 99(9), pp.1666–1672. doi: 10.2105/AJPH.2008.146209 19608959 10.2105/AJPH.2008.146209 PMC2724466

[42] Kruk ME, Mbaruku G, McCord CW, Moran M, Rockers PC, Galea S. (2009) Bypassing primary care facilities for childbirth: A population-based study in rural Tanzania. *Health Policy and Planning*, 24(4), pp.279–288. doi: 10.1093/heapol/czp011 19304785 10.1093/heapol/czp011

[43] Wagner AK, Soumerai SB, Zhang F, Ross-Degnan D. (2002) Segmented regression analysis of interrupted time series studies in medication use research. *Journal of Clinical Pharmacy and Therapeutics* 27(4): 299–309. 12174032 10.1046/j.1365-2710.2002.00430.x

[44] Rosenbaum R, Rubin D. (1985) Constructing a control group using multivariate matched sampling methods that incorporate the propensity score. *The American Statistician* 39(1): 33–38.

[45] Austin PC (2010) Optimal caliper widths for propensity-score matching when estimating differences in means and differences in proportions in observational studies. *Pharmaceut Stat* 10: 150–161. 10.1002/pst.433 PMC3120982 20925139

[46] Coca-Perraillon M. (2007) Local and Global Optimal Propensity Score Matching. Proceedings of the SAS^®^ Global Forum 2007 Conference. Cary, NC: SAS^®^ Institute Inc. Paper 185–2007.

[47] Austin PC (2009) Balance diagnostics for comparing the distribution of baseline covariates between treatment groups in propensity-score matched samples. *Statist Med* 28:3083–3107 10.1002/sim.3697 PMC3472075 19757444

[48] Yang D, Dalton JE. (2012) A unified approach to measuring the effect size between two groups using SAS^®^ Proceedings of the SAS^®^ Global Forum 2012 Conference. Cary, NC: SAS Institute Inc. Paper 335–2012

[49] Bhaskaran K, Gasparrini A, Hajat S, Smeeth L, Armstrong B. (2013) Time series regression studies in environmental epidemiology. *International Journal of Epidemiology* 42(4): 1187–95. doi: 10.1093/ije/dyt092 23760528 10.1093/ije/dyt092 PMC3780998

[50] Joharifard S, Rulisa S, Niyonkuru F, Weinhold A, Sayinzoga F, Wilkinson J, et al (2012) Prevalence and predictors of giving birth in health facilities in Bugesera District, Rwanda. *BMC Public Health* 12:1049 doi: 10.1186/1471-2458-12-1049 23217157 10.1186/1471-2458-12-1049 PMC3539866

[51] Haddad S, Fournier P. (1996) Quality, cost and utilization of health services in developing countries. A longitudinal study in Zaire. *Social Science & Medicine* 40(6): 743–753. 10.1016/0277-9536(94)00134-f 7747209

[52] Valdivia M. (2002) Public health infrastructure and equity in the utilization of outpatient health care services in Peru. *Health Policy and Planning* 17(Suppl 1): 12–19. 12477737 10.1093/heapol/17.suppl_1.12

[53] Blas E, Limbambala ME. (2001) User-payment, decentralization and health service utilization in Zambia. *Health Policy and Planning*; 16 (Suppl 2):19–28. 10.1093/heapol/16.suppl_2.19 11772987

[54] Government of Rwanda Ministry of Health (2009). Health Sector Strategic Plan (HSSP II), July 2009—June 2012. (Kigali: Ministry of Health; 2009).

[55] Ediau M, Wanyenze RK, Machingaidze S, Otim G, Olwedo A, Iriso R, et al (2013) Trends in antenatal care attendance and health facility delivery following community and health facility systems strengthening interventions in Northern Uganda. *BMC Pregnancy and Childbirth* 13:189 doi: 10.1186/1471-2393-13-189 24134717 10.1186/1471-2393-13-189 PMC3854535

