# Supplementary material for: Impact of a district-wide health center strengthening intervention on healthcare utilization in rural Rwanda: Use of interrupted time series analysis
Source: PLoS One. 2017 Aug 1;12(8):e0182418. doi: 10.1371/journal.pone.0182418 (PMC5538651; doi:10.1371/journal.pone.0182418)
Supplement: S1 File — (DOCX) [file pone.0182418.s001.docx]

**Statistical Appendix**

Our controlled interrupted time series analysis required us to develop multiple statistical models. This statistical appendix provides detailed information regarding the models that were used in this analysis, along with the modeling assumptions that were made in producing our results.

*Propensity score matching*

In order to simulate a randomized controlled trial in which intervention and non-intervention facilities were exchangeable at baseline with respect to unmeasured confounders, we used propensity score matching [1]. A propensity score is defined as the probability of treatment (in our case, probability of receiving the PHIT health center intervention) conditional on covariates. We used 380/409 (94%) publicly-run health centers in Rwanda providing care in January 2008, excluding 25 (6%) that were missing more than 4 months of data. We defined “treatment” as the 14 health centers that had not received funding from non-government partners in the intervention area prior to the PHIT intervention. Since we had a relatively few health centers that received the intervention, we chose to include population density and outpatient visits in six month intervals as covariates (Equation 1).

**Equation S1.**

$$Logit\left( \Pr\left( PHIT \right) \right)=\beta_{0}+\beta_{1}PDENS+\beta_{2}OPDB1+\beta_{3}OPDB2+\beta_{4}OPDB3+\beta_{5}OPDB4+\varepsilon$$

PHIT is a binary variable indicating whether the health center is an intervention or non-intervention facility, PDENS is a continuous variable denoting population density (number per square kilometer), OPDB1-OPDB4 are the monthly outpatient visit rates per 1,000 for the four six-month periods leading up to the intervention for each health center during the baseline period (2008-2009). Including these covariates for monthly average OPD rates for the four six-month blocks allowed us to match intervention and control health centers on baseline trend. Delivery rates were modeled in a similar fashion but were not found to be associated with probability of receiving the intervention conditional on our outpatient rates and population density, so we did not include delivery rates in our final propensity score model.

Next, we matched each of the fourteen intervention health centers to up to ten non-intervention health centers on propensity score using caliper matching within +/- 0.05 of a propensity score unit [2]. Since propensity score distributions were quite different in the intervention health centers compared to control health centers (Figure 1), we chose to include only the thirteen intervention health centers that had at least one non-intervention health center as a match.

*Controlled interrupted time series analysis*

We fit segmented regression models using the methods described by Wagner et al [CITE]. We first arranged our dataset with each row corresponding to monthly service utilization by health center, and then aggregated monthly data by intervention group (n=13 for the PHIT group, n=86 for the non-intervention group). Our time series analysis dataset contained 120 observations, one row per month for the five-year period from January 2008 – December 2012 per intervention group.

We then modeled each outcome (mean deliveries per 10,000 women, mean outpatient visits per 1,000 catchment, mean referrals for high risk pregnancies per 10,000 women, mean number of 1^st^ ANC registrations) using ARMA models. We tested for autocorrelation using Durbin-Watson statistics, autocorrelation function plots, and partial autocorrelation plots and modeled AR(p) and MA(q) processes based on these results. Given our small sample size, we chose to model seasonality using dummy variables. We provide the equations and the autocorrelation parameters for each model below (Equation 2- Equation 4).

**Equation S2. Model for monthly delivery rate following a health center strengthening intervention in 99 propensity score matched health centers**

$$delrate=\beta_{0}+\beta_{1}*time+\beta_{2}*PHIT+\beta_{3}*PHIT*time+\beta_{4}*post+\beta_{5}*post*time+\beta_{6}*post*PHIT+\beta_{7}*post*PHIT*time+\beta_{8}*seas1{+ \beta}_{9}*seas2{+ \beta}_{10}*seas3+\beta_{11}*post*seas2{+ \beta}_{12}*post*PHIT*seas2+\varepsilon$$

We used an ARMA(4,4) autoregressive moving average process to account for autocorrelation.

Delrate is the monthly rate of deliveries in a given area, β_0_ is the monthly rate of deliveries across non-intervention propensity-score matched health centers at time 1 during the pre-intervention period in Oct-Dec, time is months starting in January 2008, PHIT is the difference in monthly rate of deliveries in the intervention area compared to the non-intervention area in the pre-implementation period adjusting for season and month of follow-up, post indicates pre or post-PHIT intervention implementation, seas1-seas3 are seasonal dummy variables (January-March, April-May, June-July).

Based on these definitions, β_1_ is interpreted as the change in monthly delivery rate pre-implementation for each additional month in the non-intervention facilities. β_2_ is the difference in monthly delivery rate in intervention facilities compared to baseline facilities during the pre-implementation period. β_3_ is the residual change in monthly delivery rate pre-implementation in the PHIT facilities compared to that in the non-PHIT facilities. β_4_ is the change in level of mean delivery rate in non-intervention facilities over the post-implementation period compared to that in the pre-implementation period. β_5_ is the change in monthly delivery rate in non-intervention facilities for each additional month of follow-up post-implementation. β_6_ is the residual difference in mean delivery rates from pre- to post-implementation in PHIT facilities compared to non-intervention facilities over the post-implementation period. β_7_ is the difference in change in monthly delivery rate for each additional month post-implementation in PHIT facilities compared to non-intervention facilities. β_8_- β_10_ are the changes in mean delivery rates associated with the three month seasons compared to the final season (October-December) defined by the dummy variables.

In reviewing the plots, we hypothesized that for certain utilization metrics, some of the changes in intervention relative to comparison facilities might be concentrated in specific seasons. The terms associated with β_11_ (mean difference in monthly delivery rates in April-June compared to October-December in the post-implementation period compared to pre-implementation period) and β_12_ allow for tests of the difference in mean delivery rates in the April-June period in PHIT facilities compared to non-intervention facilities following implementation.

Our primary coefficients of interest are β_6_, β_7_ and β_12_. These coefficients tell us about the change in mean delivery rates in the post implementation period in the PHIT facilities compared to the propensity score matched non-intervention facilities.

**Equation S3. Model for monthly high risk referral rate following a health center strengthening intervention in 99 propensity score matched health centers**

$$riskreferralrate=\beta_{0}+\beta_{1}*time+\beta_{2}*PHIT+\beta_{3}*PHIT*time+\beta_{4}*post+\beta_{5}*post*time+\beta_{6}*post*PHIT+\beta_{7}*post*PHIT + \varepsilon$$

We defined riskreferralrate as the monthly referral rate (to tertiary care facilities from health centers), or the number of referrals per 10,000 women for high risk pregnancies in a study area. We omitted the seasonal dummy variables in this model because we failed to reject the null of no season variation in risk referral rate. Other beta coefficients have same definitions as above. We modeled mean referral rates using ARMA(1,1) autoregressive and moving average parameters.

**Equation S4. Model for monthly outpatient visit rate following a health center strengthening intervention in 99 propensity score matched health centers**

$$opdrate=\beta_{0}+\beta_{1}*time+\beta_{2}*PHIT+\beta_{3}*PHIT*time+\beta_{4}*post+\beta_{5}*post*time+\beta_{6}*post*PHIT+\beta_{7}*post*PHIT*time+\beta_{8}*seas1{+ \beta}_{9}*seas2{+ \beta}_{10}*seas3 + \varepsilon$$

Where opdrate is defined as the monthly outpatient visits per 1,000 catchment population in a given study area. We failed to find evidence of different outpatient visit rates in intervention compared to non-intervention facilities post-implementation in different seasons, so we omit β_11_ and β_12_ used in the delivery rate model. Other variables are defined as above.

We used ARMA(0,1) modeling to account for a moving average lag term of 1.

**Equation S5. Model for monthly 1^st^ ANC registration rate following a health center strengthening intervention in 99 propensity score matched health centers**

$$anc1rate=\beta_{0}+\beta_{1}*time+\beta_{2}*PHIT+\beta_{3}*PHIT*{time}_{i}+\beta_{4}*post+\beta_{5}*post*time+\beta_{6}*post*PHIT+\beta_{7}*post*PHIT*time+\beta_{8}*seas1{+ \beta}_{9}*seas2{+ \beta}_{10}*seas3+\beta_{11}*post*seas2+\varepsilon$$

We defined anc1rate as the monthly number of 1^st^ ANC registrations per 1,000 women in a given study area. We defined variables and beta coefficients as described above. We failed to find evidence of statistically significant intervention effect modification of the ANC 1^st^ registration rates in different seasons, so we omitted those variables from the model. We failed to find evidence of autocorrelation using Durban-Watson tests so we did not use ARMA parameters.

**Equation S6. Model for monthly 4 standard ANC visit rate following a health center strengthening intervention in 99 propensity score matched health centers**

$$anc4rate=\beta_{0}+\beta_{1}*time+\beta_{2}*PHIT+\beta_{3}*PHIT*time+\beta_{4}*post+\beta_{5}*post*time+\beta_{6}*post*PHIT+\beta_{7}*post*PHIT*time+\varepsilon$$

We defined anc4rate as the number of 4 standard ANC visits per 1,000 women per month in a given study area. We defined variables and beta coefficients as above. There was no evidence of a statistically significant difference in mean 4 standard ANC rates in intervention compared to non-intervention facilities post-implementation during the April-June season, so we omitted those variables from the model. We omitted the seasonal dummy variables because we did not have evidence of statistically significant differences in monthly 4 standard ANC visit rate by season. We modeled mean 4 standard ANC visit rates using ARMA(1) autoregressive parameters.

**Equation S7. Model for BCG vaccination rate following a health center strengthening intervention in 99 propensity score matched health centers**

$$bcgrate=\beta_{0}+\beta_{1}*time+\beta_{2}*PHIT+\beta_{3}*PHIT*time+\beta_{4}*post+\beta_{5}*post*time+\beta_{6}*post*PHIT+\beta_{7}*post*PHIT*time+\varepsilon$$

We defined bcgrate as the monthly number of BCG vaccinations per 1,000 people in a given study area. We defined variables and beta coefficients as above. There was no statistically significant difference in monthly BCG rates in intervention compared to non-intervention facilities post-implementation during the April-June season, so we did not include those betas. We modeled mean BCG vaccination rates using ARMA(2,2) autoregressive parameters.

**Equation S8. Model for DTP1 vaccination rate following a health center strengthening intervention in 99 propensity score matched health centers**

$$dtp1rate=\beta_{0}+\beta_{1}*time+\beta_{2}*PHIT+\beta_{3}*PHIT*time+\beta_{4}*post+\beta_{5}*post*time+\beta_{6}*post*PHIT+\beta_{7}*post*PHIT*time+\varepsilon$$

We defined dtp1rate as the monthly number of DTP1 vaccinations per 1,000 people in a given study area. We defined variables and beta coefficients as above. There was no statistically significant difference in monthly DTP1 rates in intervention compared to non-intervention facilities post-implementation during the April-June season, so we did not include those betas. We modeled mean DTP1 vaccination rates using ARMA(1,1) autoregressive parameters.

**Equation S9. Model for DTP3 vaccination rate following a health center strengthening intervention in 99 propensity score matched health centers**

$$dtp3rate=\beta_{0}+\beta_{1}*time+\beta_{2}*PHIT+\beta_{3}*PHIT*time+\beta_{4}*post+\beta_{5}*post*time+\beta_{6}*post*PHIT+\beta_{7}*post*PHIT*time+\varepsilon$$

We defined dtp1rate as the monthly number of DTP3 vaccinations per 1,000 people in the catchment population in intervention health centers and propensity score-matched health centers. We defined variables and beta coefficients as above. There was no statistically significant difference in mean DTP3 rates in intervention compared to non-intervention facilities post-implementation during the April-June season, so we omitted those variables. There was no statistically significant evidence of seasonality using dummy variables so we omitted those variables. We modeled mean DTP3 vaccination rates using ARMA(2,1) autoregressive parameters.

**Reference**

[1] Rosenbaum R. and Rubin D. (1985) Constructing a control group using multivariate matched sampling methods that incorporate the propensity score. *The American Statistician* 39(1): 33-38.

[2] Austin P. C. (2010) Optimal caliper widths for propensity-score matching when estimating differences in means and differences in proportions in observational studies. *Pharmaceut Stat* 10: 150-161.
