# Supplementary material for: Impact of a district-wide health center strengthening intervention on healthcare utilization in rural Rwanda: Use of interrupted time series analysis
Source: PLoS One. 2017 Aug 1;12(8):e0182418. doi: 10.1371/journal.pone.0182418 (PMC5538651; doi:10.1371/journal.pone.0182418)
Supplement: S1 Table — (DOCX) [file pone.0182418.s002.docx]

| **Analysis of Maximum Likelihood Estimates** | | | | | |
| --- | --- | --- | --- | --- | --- |
| **Parameter** | **DF** | **Estimate** | **Standard Error** | **Wald Chi-Square** | **Pr > ChiSq** |
| **Intercept** | 1 | 0.6197 | 1.0525 | 0.3467 | 0.5560 |
| **pdens** | 1 | -0.00756 | 0.00232 | 10.6231 | 0.0011 |
| **opdr1** | 1 | 0.0203 | 0.0186 | 1.1926 | 0.2748 |
| **opdr2** | 1 | -0.0518 | 0.0203 | 6.5109 | 0.0107 |
| **opdr3** | 1 | 0.0779 | 0.0313 | 6.1945 | 0.0128 |
| **opdr4** | 1 | -0.0740 | 0.0332 | 4.9866 | 0.0255 |

C-statistic: 0.861

Hosmer and Lemeshow Goodness-of-Fit Test results:

Chi-square 9.5936, df=8, p=0.29
