# Supplementary material for: Impact of a district-wide health center strengthening intervention on healthcare utilization in rural Rwanda: Use of interrupted time series analysis
Source: PLoS One. 2017 Aug 1;12(8):e0182418. doi: 10.1371/journal.pone.0182418 (PMC5538651; doi:10.1371/journal.pone.0182418)
Supplement: S2 Table — (DOCX) [file pone.0182418.s003.docx]

| **Parameter** | **Value** | **Std.Error** | **95% CI LL** | **95% CI UL** | **t-value** | **p-value** |
| --- | --- | --- | --- | --- | --- | --- |
| β0 | 26.698 | 1.912 | 22.949 | 30.446 | 13.961 | <0.0001 |
| β1 | 0.046 | 0.107 | -0.163 | 0.256 | 0.434 | 0.6654 |
| β2 | 6.609 | 2.584 | 1.544 | 11.674 | 2.558 | 0.0119 |
| β3 | -0.213 | 0.150 | -0.507 | 0.081 | -1.420 | 0.1586 |
| β4 | 1.968 | 2.241 | -2.425 | 6.360 | 0.878 | 0.3819 |
| β5 | 0.021 | 0.146 | -0.266 | 0.308 | 0.144 | 0.8861 |
| β6 | 0.668 | 3.097 | -5.402 | 6.739 | 0.216 | 0.8295 |
| β7 | 0.213 | 0.207 | -0.193 | 0.618 | 1.028 | 0.3062 |
| β8 | -0.771 | 0.778 | -2.295 | 0.754 | -0.991 | 0.324 |
| β9 | 2.631 | 1.063 | 0.547 | 4.715 | 2.474 | 0.0149 |
| β10 | -1.335 | 0.726 | -2.759 | 0.089 | -1.838 | 0.0688 |
| β11 | -2.646 | 1.292 | -5.177 | -0.114 | -2.048 | 0.043 |
| β12 | 3.186 | 1.487 | 0.272 | 6.099 | 2.143 | 0.0344 |

**Correlation Parameter Estimates**

**Autocorrelation parameters:**

| Phi1 | Phi2 | Phi3 | Phi4 | Theta1 | Theta2 | Theta3 | Theta4 |
| --- | --- | --- | --- | --- | --- | --- | --- |
| 0.1612 | 0.5534 | 0.4513 | -0.7858 | 0.3850 | -0.0573 | -0.4541 | 0.4084 |
