# Supplementary material for: Impact of a district-wide health center strengthening intervention on healthcare utilization in rural Rwanda: Use of interrupted time series analysis
Source: PLoS One. 2017 Aug 1;12(8):e0182418. doi: 10.1371/journal.pone.0182418 (PMC5538651; doi:10.1371/journal.pone.0182418)
Supplement: S3 Table — (DOCX) [file pone.0182418.s004.docx]

|  | Value | 95% LL | 95% UL | Std.Error | t-value | p-value |
| --- | --- | --- | --- | --- | --- | --- |
| β0 | 2.181 | 1.8724 | 2.4890 | 0.157 | 13.863 | <0.0001 |
| β1 | -0.023 | -0.0417 | -0.0051 | 0.009 | -2.512 | 0.0134 |
| β2 | -0.824 | -1.2600 | -0.3880 | 0.222 | -3.704 | 0.0003 |
| β3 | -0.014 | -0.0395 | 0.0123 | 0.013 | -1.030 | 0.3054 |
| β4 | 0.055 | -0.3335 | 0.4430 | 0.198 | 0.276 | 0.7828 |
| β5 | 0.024 | -0.0002 | 0.0486 | 0.012 | 1.941 | 0.0547 |
| β6 | 0.035 | -0.5136 | 0.5846 | 0.280 | 0.127 | 0.8994 |
| β7 | 0.027 | -0.0071 | 0.0620 | 0.018 | 1.559 | 0.1218 |

**Correlation parameters**

| Phi1 | Theta1 |
| --- | --- |
| 0.1184706 | 0.354718 |
