# Supplementary material for: Impact of a district-wide health center strengthening intervention on healthcare utilization in rural Rwanda: Use of interrupted time series analysis
Source: PLoS One. 2017 Aug 1;12(8):e0182418. doi: 10.1371/journal.pone.0182418 (PMC5538651; doi:10.1371/journal.pone.0182418)
Supplement: S4 Table — (DOCX) [file pone.0182418.s005.docx]

| **Parameter** | **Value** | **95% CI LL** | **95% CI UL** | **Std.Error** | **t-value** | **p-value** |
| --- | --- | --- | --- | --- | --- | --- |
| β0 | 50.573 | 38.399 | 62.746 | 6.211 | 8.142 | <0.0001 |
| β1 | 0.350 | -0.319 | 1.018 | 0.341 | 1.026 | 0.3073 |
| β2 | -13.257 | -29.101 | 2.588 | 8.084 | -1.640 | 0.1039 |
| β3 | 0.523 | -0.420 | 1.465 | 0.481 | 1.086 | 0.2797 |
| β4 | 1.260 | -13.035 | 15.556 | 7.294 | 0.173 | 0.8631 |
| β5 | -0.805 | -1.690 | 0.080 | 0.451 | -1.783 | 0.0773 |
| β6 | 6.645 | -13.522 | 26.812 | 10.290 | 0.646 | 0.5198 |
| β7 | 0.073 | -1.177 | 1.324 | 0.638 | 0.115 | 0.9086 |
| β8 | 1.760 | -4.553 | 8.072 | 3.221 | 0.546 | 0.5859 |
| β9 | 14.595 | 7.953 | 21.237 | 3.389 | 4.307 | <0.0001 |
| β10 | -3.549 | -9.677 | 2.579 | 3.127 | -1.135 | 0.2588 |

**Correlation Parameter Estimates:**

Theta1

0.4520761
