# Supplementary material for: Impact of a district-wide health center strengthening intervention on healthcare utilization in rural Rwanda: Use of interrupted time series analysis
Source: PLoS One. 2017 Aug 1;12(8):e0182418. doi: 10.1371/journal.pone.0182418 (PMC5538651; doi:10.1371/journal.pone.0182418)
Supplement: S5 Table — (DOCX) [file pone.0182418.s006.docx]

|  | **Value** | **95% LL** | **95% UL** | **Std.Error** | **t-value p-** | **value** |
| --- | --- | --- | --- | --- | --- | --- |
| β0 | 6.0780 | 5.5612 | 6.5947 | 0.2636 | 23.0541 | <0.0001 |
| β1 | -0.0597 | -0.0876 | -0.0318 | 0.0142 | -4.1951 | 0.0001 |
| β2 | -0.7667 | -1.4198 | -0.1135 | 0.3332 | -2.3006 | 0.0233 |
| β3 | 0.0188 | -0.0205 | 0.0582 | 0.0201 | 0.9372 | 0.3508 |
| β4 | 0.8151 | 0.1982 | 1.4320 | 0.3148 | 2.5897 | 0.0109 |
| β5 | 0.0574 | 0.0214 | 0.0933 | 0.0183 | 3.1281 | 0.0023 |
| β6 | 0.2668 | -0.6015 | 1.1352 | 0.4430 | 0.6023 | 0.5483 |
| β7 | -0.0167 | -0.0676 | 0.0341 | 0.0259 | -0.6449 | 0.5203 |
| β8 | 0.9166 | 0.6040 | 1.2292 | 0.1595 | 5.7464 | <0.0001 |
| β9 | 0.3206 | 0.0090 | 0.6323 | 0.1590 | 2.0167 | 0.0462 |
| β10 | -0.2704 | -0.5799 | 0.0390 | 0.1579 | -1.7130 | 0.0896 |
