# Supplementary material for: Impact of a district-wide health center strengthening intervention on healthcare utilization in rural Rwanda: Use of interrupted time series analysis
Source: PLoS One. 2017 Aug 1;12(8):e0182418. doi: 10.1371/journal.pone.0182418 (PMC5538651; doi:10.1371/journal.pone.0182418)
Supplement: S6 Table — (DOCX) [file pone.0182418.s007.docx]

|  | **Value** | **95% LL** | **95% UL** | **Std.Error** | **t-value p-** | **value** |
| --- | --- | --- | --- | --- | --- | --- |
| β0 | 0.4125 | 0.27362 | 0.55129 | 0.0708 | 5.8229 | <0.0001 |
| β1 | 0.0082 | 0.00019 | 0.01615 | 0.0041 | 2.0062 | 0.0472 |
| β2 | -0.1787 | -0.37508 | 0.01760 | 0.1002 | -1.7843 | 0.0771 |
| β3 | -0.0052 | -0.01646 | 0.00611 | 0.0058 | -0.8986 | 0.3708 |
| β4 | 0.0234 | -0.13067 | 0.17740 | 0.0786 | 0.2973 | 0.7668 |
| β5 | 0.0143 | 0.00302 | 0.02559 | 0.0058 | 2.4851 | 0.0144 |
| β6 | 0.0325 | -0.18535 | 0.25032 | 0.1111 | 0.2923 | 0.7706 |
| β7 | 0.0027 | -0.01330 | 0.01862 | 0.0081 | 0.3267 | 0.7445 |

**Correlation parameters**

| Phi1 |
| --- |
| 0.5645208 |
