# Supplementary material for: Impact of a district-wide health center strengthening intervention on healthcare utilization in rural Rwanda: Use of interrupted time series analysis
Source: PLoS One. 2017 Aug 1;12(8):e0182418. doi: 10.1371/journal.pone.0182418 (PMC5538651; doi:10.1371/journal.pone.0182418)
Supplement: S7 Table — (DOCX) [file pone.0182418.s008.docx]

|  | **Value** | **95% LL** | **95% UL** | **Std.Error** | **t-value** | **p-value** |
| --- | --- | --- | --- | --- | --- | --- |
| β0 | 2.7663 | 2.4039 | 3.1287 | 0.1849 | 14.9608 | <.0001 |
| β1 | -0.0090 | -0.0276 | 0.0096 | 0.0095 | -0.9467 | 0.3459 |
| β2 | -0.0328 | -0.5194 | 0.4539 | 0.2483 | -0.1319 | 0.8953 |
| β3 | -0.0005 | -0.0267 | 0.0258 | 0.0134 | -0.0353 | 0.9719 |
| β4 | -0.3853 | -0.6612 | -0.1095 | 0.1407 | -2.7377 | 0.0072 |
| β5 | 0.0221 | -0.0066 | 0.0509 | 0.0147 | 1.5097 | 0.134 |
| β6 | 0.3505 | -0.0390 | 0.7401 | 0.1988 | 1.7637 | 0.0806 |
| β7 | -0.0010 | -0.0415 | 0.0396 | 0.0207 | -0.0472 | 0.9625 |
| β8 | 0.1020 | -0.0586 | 0.2627 | 0.0820 | 1.2446 | 0.2159 |
| β9 | 0.2501 | 0.0399 | 0.4602 | 0.1072 | 2.3321 | 0.0215 |
| β10 | -0.0446 | -0.1982 | 0.1091 | 0.0784 | -0.5687 | 0.5707 |

**Correlation parameters**

| Phi1 | Phi2 | Theta1 | Theta2 |
| --- | --- | --- | --- |
| 1.4654522 | -0.7591 | -1.31099 | 0.939104 |
