# Supplementary material for: Impact of a district-wide health center strengthening intervention on healthcare utilization in rural Rwanda: Use of interrupted time series analysis
Source: PLoS One. 2017 Aug 1;12(8):e0182418. doi: 10.1371/journal.pone.0182418 (PMC5538651; doi:10.1371/journal.pone.0182418)
Supplement: S8 Table — (DOCX) [file pone.0182418.s009.docx]

|  | **Value** | **95% LL** | **95% UL** | **Std.Error** | **t-value** | **p-value** |
| --- | --- | --- | --- | --- | --- | --- |
| β0 | 2.9097 | 2.4185 | 3.4009 | 0.2506 | 11.6102 | <0.0001 |
| β1 | -0.0155 | -0.0421 | 0.0112 | 0.0136 | -1.1362 | 0.2584 |
| β2 | -0.1988 | -0.8721 | 0.4744 | 0.3435 | -0.5789 | 0.5639 |
| β3 | 0.0148 | -0.0229 | 0.0525 | 0.0192 | 0.7705 | 0.4427 |
| β4 | -0.1207 | -0.5910 | 0.3497 | 0.2400 | -0.5029 | 0.6161 |
| β5 | 0.0253 | -0.0140 | 0.0645 | 0.0200 | 1.2605 | 0.2102 |
| β6 | 0.0082 | -0.6554 | 0.6718 | 0.3386 | 0.0242 | 0.9807 |
| β7 | -0.0124 | -0.0678 | 0.0431 | 0.0283 | -0.4373 | 0.6628 |
| β8 | -0.0114 | -0.1703 | 0.1474 | 0.0810 | -0.1412 | 0.888 |
| β9 | 0.1050 | -0.0672 | 0.2772 | 0.0879 | 1.1950 | 0.2347 |
| β10 | 0.1149 | -0.0352 | 0.2649 | 0.0766 | 1.5007 | 0.1363 |

**Correlation parameters**

| Phi1 | Theta1 |
| --- | --- |
| 0.752287 | -0.27169 |
