# Supplementary material for: Impact of a district-wide health center strengthening intervention on healthcare utilization in rural Rwanda: Use of interrupted time series analysis
Source: PLoS One. 2017 Aug 1;12(8):e0182418. doi: 10.1371/journal.pone.0182418 (PMC5538651; doi:10.1371/journal.pone.0182418)
Supplement: S9 Table — (DOCX) [file pone.0182418.s010.docx]

|  | **Value** | **95% LL** | **95% UL** | **Std.Error** | **t-value** | **p-value** |
| --- | --- | --- | --- | --- | --- | --- |
| β0 | 2.8895 | 2.4841 | 3.2949 | 0.2068 | 13.9700 | <.0001 |
| β1 | -0.0100 | -0.0328 | 0.0129 | 0.0117 | -0.8549 | 0.3944 |
| β2 | -0.2378 | -0.8111 | 0.3356 | 0.2925 | -0.8128 | 0.4181 |
| β3 | 0.0251 | -0.0072 | 0.0574 | 0.0165 | 1.5209 | 0.1311 |
| β4 | -0.2387 | -0.6501 | 0.1727 | 0.2099 | -1.1373 | 0.2578 |
| β5 | 0.0202 | -0.0131 | 0.0535 | 0.0170 | 1.1872 | 0.2376 |
| β6 | -0.3411 | -0.9229 | 0.2406 | 0.2968 | -1.1493 | 0.2529 |
| β7 | -0.0199 | -0.0670 | 0.0272 | 0.0240 | -0.8286 | 0.4091 |

**Correlation parameters**

| Phi1 | Phi2 | Theta1 |
| --- | --- | --- |
| -0.2663 | 0.613575 | 0.766741 |
