# Supplementary figures and images for: Impact of a district-wide health center strengthening intervention on healthcare utilization in rural Rwanda: Use of interrupted time series analysis
Source: PLoS One. 2017 Aug 1;12(8):e0182418. doi: 10.1371/journal.pone.0182418 (PMC5538651; doi:10.1371/journal.pone.0182418)

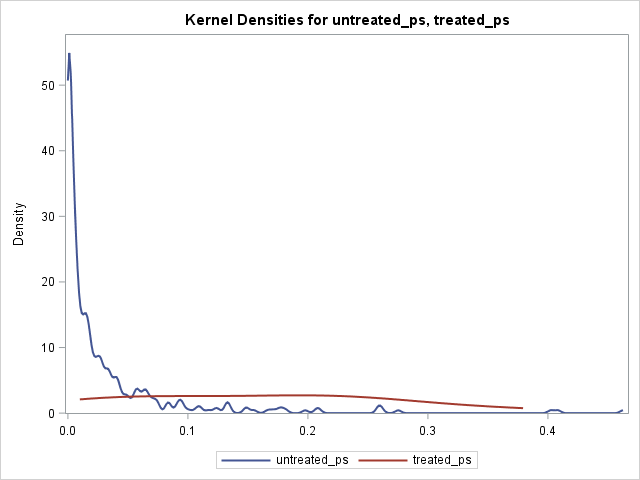

Supplement: S1 Fig — (TIF) [file pone.0182418.s011.tif]
